# Short-Term High Intensity Plyometric Training Program Improves Strength, Power and Agility in Male Soccer Players

**DOI:** 10.2478/hukin-2013-0002

**Published:** 2013-03-28

**Authors:** Márk Váczi, József Tollár, Balázs Meszler, Ivett Juhász, István Karsai

**Affiliations:** 1 Institute of Physical Education and Sport Sciences, University of Pécs, Hungary.; 2 Doctoral School of Health Sciences, University of Pécs, Hungary.; 3 Institute of Physiotherapy and Nutritional Sciences, University of Pécs, Hungary.

**Keywords:** knee extensors, depth jump, dynamometer, unilateral, plyometrics

## Abstract

The aim of the present study was to investigate the effects of a short-term in-season plyometric training program on power, agility and knee extensor strength. Male soccer players from a third league team were assigned into an experimental and a control group. The experimental group, beside its regular soccer training sessions, performed a periodized plyometric training program for six weeks. The program included two training sessions per week, and maximal intensity unilateral and bilateral plyometric exercises (total of 40 – 100 foot contacts/session) were executed. Controls participated only in the same soccer training routine, and did not perform plyometrics. Depth vertical jump height, agility (Illinois Agility Test, T Agility Test) and maximal voluntary isometric torque in knee extensors using Multicont II dynamometer were evaluated before and after the experiment. In the experimental group small but significant improvements were found in both agility tests, while depth jump height and isometric torque increments were greater. The control group did not improve in any of the measures. Results of the study indicate that plyometric training consisting of high impact unilateral and bilateral exercises induced remarkable improvements in lower extremity power and maximal knee extensor strength, and smaller improvements in soccer-specific agility. Therefore, it is concluded that short-term plyometric training should be incorporated in the in-season preparation of lower level players to improve specific performance in soccer.

## Introduction

Plyometric training (PT) is popular among individuals involved in dynamic sports, and plyometric exercises such as jumping, hopping, skipping and bounding are executed with a goal to increase dynamic muscular performance (Impellizzeri et al., 2008; Wilson et al., 1993; Wilson et al., 1996). In these exercises muscles undergo a rapid elongation followed by an immediate shortening (stretch-shortening contraction), utilizing the elastic energy stored during the stretching phase (Cavagna, 1977).

PT has been applied in numerous studies, and there is a general consensus that it improves sport specific skills such as agility (Miller et al., 2006) and vertical jump performance, common measures of muscle power (Markovic, 2007). In soccer rapid movements such as acceleration and deceleration of the body, changes of direction, as well as jumps are often performed and high level of dynamic muscular performance is required at all levels of training status. In investigations mostly elite soccer players were recruited to demonstrate the effects of PT on muscular performance (Chimera et al., 2004; Ronnestad et al., 2008; Sedano Campo et al., 2009), and results are often conflicting. For example in division I female soccer players 6 weeks of PT either improved (Sedano Campo et al., 2009) or did not improve (Chimera et al., 2004) vertical jump height. In a different study male professionals were trained for 7 weeks and it was found that PT combined with strength training improved various dynamic measures, but not vertical jump performance (Ronnestad et al., 2008). Inconsistencies in studies can be attributed to several factors, such as gender, training status, methods of testing, different types of apparatus, and differences in duration, intensity, and the types of the exercises used in the training program (Markovic, 2007).

One problem with regard to the PT studies is that many times in short-term programs either low intensity (Rubley et al., 2011) or low impact (Miller et al., 2006) exercises are performed, or intensity is not reported at all (Miller et al., 2006; Ebben et al., 2010). In a study by Chimera et al. (2004) plyometric exercises with 30s duration or with 30 to 70 repetitions were performed continuously with a possibility of improperly inducing high fatigue in participants. Furthermore, mostly bilateral (two-leg) exercises were performed (Impellizzeri et al., 2008; Sedano Campo et al., 2009; Perez-Gomez et al., 2008; Chelly et al., 2010; Thomas et al., 2009), while including more intensive unilateral (single-leg jump) exercises into short-term PT programs may be beneficial with a goal of rapid strength gain. Makaruk et al. (2011) for example demonstrated that in untrained women unilateral jump training improved power and vertical jump height in a shorter period, compared with bilateral jump training. Though unilateral exercises have been extensively used by track and field athletes for decades in various age groups and levels to maximize sprint speed, jumping height and distance, and has also been used among recreationally trained participants in scientific investigation (Malisoux et al., 2006), there is a lack of evidence that these exercises are used in lower level soccer players. Much less information is available on the effectiveness of PT in these individuals (Thomas et al., 2009), while improving dynamic muscular performance is also highly recommended for lower class teams to focus on. Finally, single-leg plyometric movements into lateral directions are rarely included in programs, or if included, maximum intensity was not required or at least was not reported by the authors (Miller et al., 2006).

The aim of the present study was to investigate the effects of a six-week-long periodized PT program on agility and power among male adult soccer players, using high impact unilateral and bilateral plyometric exercises. As knee extensors are highly involved in soccer movements (kicking, jumping, changes of direction), it was also our purpose to observe maximum strength changes in the knee extensor muscles. Therefore, we tested the hypothesis that the six-week-long PT program improves agility, depth vertical jump height and knee extensor strength.

## Material and Methods

### 

#### Participants

Twenty-four males were recruited and randomly assigned to a plyometric training (PL, n = 12) (age = 21.9 ± 1.7 years; body mass = 75.9 ± 2.7 kg; body height = 180.1 ± 4.0 cm) or control (n = 12) group (age = 22.7 ± 1.4 years; body mass = 78.6 ± 3.1 kg; body height = 180.6 ± 3.7 cm). Participants were selected from two Hungarian third league soccer teams, and have been training for at least 7 years. During the experiment participants from both groups were involved in 3 to 4 regular soccer training sessions per week, and participated in one game on the weekends. All participants have previously done unilateral or bilateral plyometric exercises. Before any training and testing an oral explanation of the experimental procedures was given to all participants. After this a written informed consent form was signed according to the Declaration of Helsinki and subjects agreed to participate in the experiment which was approved by the University Ethics Committee. All participants were familiarized with the test exercises at least one week before the beginning of the experiment. None of the participants reported current injuries of the spine or the lower extremities and no injuries occurred during the experiment.

### Procedures

The experiment consisted of two test sessions (pre and post-exercise test) and a PT intervention. Pre-exercise tests were preformed three days before the beginning of training and included both laboratory and field tests to evaluate lower extremity strength, power and agility. A six-week-long periodized PT program was applied, followed by post-exercise tests, three days after the last training session. During the experiment all participants continued their regular soccer training routine, that was identical for every participant, except that group PL participated in additional PT program, and controls did the exercise tests only. All testing sessions began with a standardized warm-up: five minutes aerobic warm up (jogging) followed by stretching of the lower extremity muscles. One familiarization trial of each of the exercise tests was executed with submaximal intensity before the actual measurement. For training the participants warmed up with their usual routine. This comprised ten minutes jogging, stretching, performing 8 to 10 running drills into different directions, and 4 to 6 submaximal running strides. The plyometric drills in the experimental group were always executed immediately after the warm-up, before any other training tasks were performed on the given day.

### Agility

It has been previously suggested that PT improves sport specific agility in sports where sudden movements (accelerations, stops and direction changes) are required (Yap and Brown, 2000). Two specific agility tests were performed in the present investigation. The T agility test (TAT) was applied to measure agility during direction changes such as forward sprints, left and right shuffles, and backpedalling (Miller et al., 2006). In this test three cones were set five meters apart on a straight line and a fourth cone was placed ten meters from the middle cone, forming a T shape. The Illinois agility test (IAT) was used to measure agility during sprints including direction changes without stopping, and running at different angles (Miller et al., 2006). Additional information about these tests has been reported by Miller et al. (2006). Participants performed two trials of each of the agility tests with five minutes recovery between trials, and ten minutes recovery between test types. The best time of the two trials was considered for later analysis. Times to complete the agility tests were measured every time by the same three assisting people using a stop watch. The average of the times measured by the three assistants was used for statistics. Until the end of the experiment the experimental status of the participants (PL or control) was unknown for all assistants.

### Depth vertical jump performance

Depth vertical jump height (DVJ) was measured to evaluate multi-joint power generating ability in the lower extremities. Participants were standing next to a wall extending one arm to touch the highest point possible while remaining flat-footed, to record standing reach height. After this procedure participants dropped from a 22cm platform and performed a maximal effort double-leg vertical jump using arm swing. The test was performed using the chalk method (Twist and Eston, 2005) by rubbing chalk on the fingers of the dominant hand and reaching to leave a mark on the wall as high as possible (while remaining flat-footed), after which followed a proper form to jump and place a chalk mark as high as possible on the wall. Instructions were given to quickly reverse the downward movement of the body to an upward movement in order to minimize the contact time with ground upon landing, and maximize take-off velocity. The difference between standing reach height and vertical jump height was calculated for every trial. Three trials were allowed and the best DVJ was recorded for further analysis. There was one minute rest between trials.

### Maximal voluntary isometric torque

A custom-built computerized isokinetic dynamometer (Multicont II, Mediagnost, Budapest and Mechatronic Ltd., Szeged, Hungary), described in detail previously (Váczi et al., 2011), was used for the evaluation of knee extensor static strength. Participants were seated in the dynamometer’s seat. The torso was stabilized with shoulder harnesses and both thighs were secured with rubberized Velcro straps to the dynamometer’s seat. The shin of the dominant lower extremity, above the ankle, was fastened with a strap to the lever of a servo motor (MA-10, maximal velocity: 6000 rpm). The apparent knee joint centre of rotation was aligned with the centre of rotation of the lever arm. Participants performed three trials of maximal isometric contraction at 60° of knee angle position. It was required to slowly generate the highest possible torque with the knee extensors. From torque-time maximal voluntary isometric torque (MVC) was determined. Knee extensor torque was assessed only in the dominant (kicking) leg.

### Plyometric training

Specific details of the PT program are presented in Table 1. A six-week-long training program was applied in group PL with similar periodization described previously by Miller et al. (2006). The first two weeks (W1 and W2) were a preparatory phase, followed by three more weeks (W3–W5) with increased volume, and one week (W6) with decreased volume to taper. Training sessions were performed twice a week (Tuesdays and Thursdays) as recommended by others to allow time for regeneration (Adams et al., 1992). Unlike in other plyometric training routines the specificity of the present program was that beside double-leg jumps, high intensity single-leg exercises into both sagittal and lateral directions were also included, with a goal of rapid gain in agility. Participants were instructed to minimize ground contact and to maximize jumping height (in hurdle, cone and depth jumps) or distance (in forward hops). It has been previously suggested that time between eccentric and concentric actions (coupling time) must be as short as possible as the shorter the coupling time, the greater the release of stored energy (Komi, 1984). A conditioning specialist supervised every training session to maximize safety by instructing proper technique and to motivate participants for maximal effort.

### Analyses

Means and standard deviations as descriptive statistics were calculated for the measured variables. All variables were tested for normality. To identify a significant group by time interactions, 2×2 (group by time) analyses of variance with repeated measures were used for each dependent variable. When significant interaction was found Tukey’s post hoc analysis was performed for pairwise comparisons. The statistical significance was set at *p* < 0.05.

## Results

All variables were normally distributed as suggested by the Kolmogorov-Smirnov test results (*p* > 0.05). The effect size for the selected variables ranged between 0.42 and 0.90. ANOVA revealed significant group by time interaction effects for TAT (F1,22 = 8.49, *p* < 0.01), IAT (F1,22 = 5.09, *p* < 0.05), DVJ (F1,22 = 16.8, *p* < 0.01), and MVC (F1,8 = 11.2, *p* < 0.01). Pre- to post-training changes for the two groups are presented in Table 2. In the PL group post-hoc tests revealed the greatest improvement in DVJ (9%) (*p* < 0.05), and MVC increased (7%) from pre- to post-training (*p* < 0.05). Small but significant improvement was found in TAT and IAT (2.5% and 1.7%, respectively) (*p* < 0.05). In controls there was no change over time in any of the measured variables.

## Discussion

In the present investigation we hypothesized that six weeks of plyometric training, comprising both unilateral and bilateral maximal intensity exercises, would produce improvements in power, strength, and agility in third league male soccer players. It was found that the training program significantly improved depth vertical jump performance, agility, and isometric knee extensor strength.

### Depth vertical jump performance

The greatest improvement in the experimental group was found in depth vertical jump performance. A change of 9% indicates that significant adaptation in leg power has occurred, showing the benefits of maximal intensity PT training. The majority of the studies demonstrate positive changes in countermovement jump tests, but less data is available on depth jump performance, a more specific measure of leg power in sports, where high impact forces are present during movements. Chimera et al. (2004) reported 4% improvement in depth jump performance among athletes, while Young et al. (2009) found 7–9% improvement in non-athletes, after six weeks of PT. Considering our participants’ competitive training status we conclude that 9% change in depth jump performance is remarkable, and greater improvement has been previously noticed only in non-athletes, and after longer (8 and 15 weeks) training periods (Lehance et al., 2005; Kyrolainen et al., 2005). Such change in only six weeks in our study strengthens previous evidence that unilateral plyometric exercises can improve vertical jump performance in a short period of time (Makaruk et al., 2011). As previously suggested, positive changes in power, after such a short training period as in the present study, can be associated with the neural components of adaptation: specifically with an increased neural drive to the agonist muscles and changes in the muscle activation strategies (i.e. improved intermuscular coordination), or changes in the mechanical characteristics of the muscle-tendon complex (Markovic and Mikulic, 2010). These neurophysiological changes together may improve the ability to store and release elastic energy during the stretch-shortening cycle. Specifically, upon landing after a depth jump, an increased level of pre-activation enables the muscle sarcomeres to maintain their length, while the tendons keep elongating and store elastic energy (Kopper et al., 2012). In other words, in the eccentric phase of the depth jump the whole muscle lengthens, but the contractile apparatus maintains its length due to high activation.

### Agility

When sprint performance was evaluated after PT, results from different studies were contradicting. In male professional soccer players, Ronnestad et al. (2008) found improvements in acceleration, peak running velocity and overall sprint time (40m sprint) after 7 weeks combined PT and strength training. In contrast, Perez-Gomez et al. (2008) found no change in either short (5–30m) or long (300m) sprints after 6 weeks of a similar training program in physical education students. Similarly, Impellizzeri et al. (2008) demonstrated no effect of 4 weeks PT on 10 or 20m sprint time in amateur soccer players, probably because of the short training period.

Measuring agility could be more specific in the evaluation of the physical status of soccer players, as acceleration and deceleration, sudden stops and direction changes occur frequently during games. In the present investigation, two types of tests were applied to evaluate changes in agility. Slight but significant improvements were observed both in the T agility (2.5%) and in the Illinois agility (1.7%) tests. Fewer studies examined the effects of PT on specific agility, but results are more consistent in contrast with those obtained from sprint tests. Thomas et al. (2009) found that despite that sprint time was unchanged, six weeks of PT significantly improved agility (9%) in semiprofessional adolescent soccer players. The greatest improvement in agility (10%) was found in children soccer players after 8 weeks of PT (Meylan and Malatesta, 2009). Miller et al. (2006) found 5 and 3% improvements in the T agility and Illinois agility tests, respectively, after 6 weeks of PT. These improvements are greater than those obtained in the present study, however, making a comparison is difficult as training status of the participants is not reported in the study by Miller et al. (2006). From these research data, in agreement with our results, a conclusion can be made that a short-term PT program is effective for improving soccer specific agility, though previous studies show that sprint performance is not always enhanced in such a short interval. The magnitude of improvement in agility, on one hand, may be influenced by the training status or age of the participants, demonstrating greater agility enhancement in younger individuals versus adults. Another influencing factor can be the type of the agility test, specifically the time to complete the test. Meylan and Malatesta (2009) used a 10m test with four 60-degree turns around a pole, and participants completed the test in an average of 4.5 seconds. Thomas et al. (2009) used the 505 agility test (Ellis et al., 2000) and the average time to complete this test for the participants was also small (2.7 seconds). Both in the study by Miller et al. (2006) and in ours the magnitude of changes in the two agility tests was smaller than in those mentioned previously. It is possible that these short term PT programs, despite that significant changes are noticed in power, show less improvement in anaerobic capacity, or muscle efficiency, the ability to maintain high mechanical work with less metabolic costs. Considering that the T agility and Illinois agility tests required ∼11 and ∼14 seconds to complete, respectively, during these tests not only the ATP-PC system, but the glycolitic energy system is also utilized, and this could be the explanation why improvements are smaller compared with the agility tests that require less time for execution.

Overall, improvements in agility after plyometric training can be attributed to neural adaptation, specifically to increased intermuscular coordination. Previous research also demonstrated increased proprioception after plyometric training (Myer et al., 2006). In our training protocol we also applied single leg jumps in lateral directions with the goal to increase joint stability and proprioception, important factors in performance when agility tasks are performed with stops and direction changes. Though, even with these laterally performed jump drills, improvements were less in our experiment than in others’, the ∼0.30 second absolute change is remarkable in only six weeks in these third league soccer players.

### Knee extensor strength

Effects of PT on lower extremity strength have been characterized in various populations, and in a recent meta-analysis a conclusion was made that PT improved one repetition maximum measured in isometric or slow velocity contractions in leg muscles (Sáez-Sáez de Villarreal et al., 2010). Arazi and Asadi (2011) reported 15% gain in leg press in semi-professional male basketball players, following 8 weeks PT. Though, it has been suggested that leg muscle strength is an important factor in kicking performance (De Profte et al., 1988), limited data are available on the effects of PT on knee extensor strength in soccer players. Maximal isometric knee extensor strength improved 7% in our participants after a program in which only explosive, and no heavy resistance training exercises were used. Similar change was found by Perez-Gomez et al. (2008) after 6 weeks training, but both plyometric and weight lifting exercises were used. Ronnestad et al. (2008) demonstrated 25% gain in 1RM squat in male Norwegian first league soccer players after 7 weeks of training, but in the program strength and plyometric exercises were combined, therefore their results are not surprising. A suggestion has been made previously that subjects in either good or poor physical condition, benefit equally from plyometric work (Sáez-Sáez de Villarreal et al., 2010). Also training volume of less than 10 weeks and with more than 15 sessions, as well as the inclusion of high-intensity programs, with more than 40 jumps per session, were the strategies that seem to maximize the probability to obtain significantly greater improvements in leg muscle strength (Sáez-Sáez de Villarreal et al., 2010). Knee extensor strength improvement in our subjects suggests increased motor unit synchronization. Measuring maximal isometric torque evaluates neuromuscular adaptation regardless of motor unit and fiber type involvement. It is possible that our plyometric training program selectively trained the fast twitch fibers and this contributed to a 7% increase in MVC, which is a measure of static contractility of all fibers. Magnitude of strength changes could also be influenced by the duration of the training program. Notably, one cannot exclude that longer programs do not increase muscle size, since plyometric training alone has been proved to induce significant hypertrophy (Malisoux et al., 2006), and if combined with resistance training it is possible that strength changes are greater (Ronnestad et al., 2008).

Discrepancies in the outcomes of the PT programs could be explained with the differences in the training intensity, volume, and whether PT was combined with strength training or not. In contrast to other studies that also used heavy resistance exercises (Ronnestad et al., 2008; Perez-Gomez et al., 2008), in the present training protocol only maximal intensity explosive exercises were utilized with an objective to achieve a complex improvement in physical performance required for soccer, such as jumping ability, knee extensor strength and agility. We demonstrated that plyometric training alone is sufficient to increase physical performance required in soccer. One unique characteristics of the present training program is that training volume was relatively low compared with those in other studies (Miller et al., 2006; Chimera et al., 2004; Sedano Campo et al., 2009). With as short as 10 to 20 minutes training duration, and with an average of 70 foot contacts per session our participants achieved similar gains in jumping performance than that described by Seadano Campo et al. (2009) using an average of 90 foot contacts. Miller et al. (2006) applied an average of 120 contacts per session, and this increased agility more than in our protocol. Though training status of Miller’ participants was not reported, based on their agility test times we assume that our participants were in better physical condition, therefore improvements are difficult to compare. Furthermore, despite that an extreme PT program with approximately 270–640 foot contacts/session were performed for six weeks, less improvement (5.8%) was reported in vertical jump height (Chimera et al., 2004), compared with our results.

Another unique characteristics of the training described in the present study is that maximal intensity unilateral exercises were also included, and they were also performed into lateral directions. These high impact jumping and hopping exercises seem to have a significant, short-term effect on depth vertical jump performance (9%) and maximal knee extensor strength (7%). It is still interesting that despite such remarkable improvement in depth vertical jump and knee extensor strength, gains in agility were relatively small, and it is possible that a combination of sprint and PT would better enhance agility. It is also possible that optimal volume, training frequency and duration would be different for improving particular performance measures in soccer (jumping ability, sprint time, agility, strength). Furthermore, using agility tests that require less time to complete, greater improvements could be noticed.

Power, strength, and agility are important performance factors in sports where dynamic movements, such as jumps, sprints, stops, direction changes, and lateral movements are performed. Individual quality of these factors highly contributes to team performance on the field. It has been shown that elite soccer teams benefit from plyometric training, however attention must also be paid to lower class players. In European countries the number of teams in the national first league is 10 to 20, while in lower leagues this number can be manifold greater, and improving soccer specific performance is challenging for these teams. Though these teams execute much less training sessions per week when compared with professionals, such low volume and high intensity conditioning programs, with only 10 to 20 minutes duration, can fit into their training regimen. Results of the present study indicate that 6 weeks of plyometric training consisting of maximal intensity unilateral and bilateral exercises induced remarkable improvements in lower extremity power and maximal knee extensor strength, and smaller improvements in soccer-specific agility in third league male soccer players, and these performance measures are all important factors of improving the quality of soccer games. In agreement with other authors examining elite soccer players, it is considered to be important to include short-term plyometric programs in in-season preparation in order to improve complex soccer specific dynamic performance.

## Figures and Tables

**Table 1:** A detailed description, including number of sets (first) and repetitions (second), of the six-week-long combined single and double-leg plyometric training program

	**W1 to W2**		**W3 to W5**		**W6**	

*Plyometric exercise*	Mon	Thu	Mon	Thu	Mon	Thu
		
Double-leg hurdle jump (90cm)	4×5	-	6×5	-	3×5	-
Single-leg lateral cone jump (35cm)	3×10	-	4×10	-	2×10	-
Single leg toward hop	3×5	-	4×5	-	2×5	-
Double-leg depth jump (55cm)	-	4×5	-	6×5	-	2×5
Double-leg lateral cone jump (35cm)	-	4×5	-	6×5	-	2×5
Single-leg hurdle jump (35cm)	-	3×10	-	4×10	-	2×10

*Total unilateral foot contacts/leg/session*	45	30	60	40	30	20
*Total bilateral foot contacts/session*	20	40	30	60	15	20

W= week

**Table 2:** Effects of the six-week-long maximal intensity plyometric training program on agility, depth vertical jump performance, and maximal voluntary torque (MVC) of the knee extensors. *Significant difference between pre- and post-test after Tukey post-hoc analysis (p < 0.05)

	Plyometric (n = 12)				Control (n = 12)			
	Pre-test		Post-test		Pre-test		Post-test	
	
*Performance Variables*	*Mean*	*SD*	*Mean*	*SD*	*Mean*	*SD*	*Mean*	*SD*

T-Sprint Test (s)	11.72	0.90	11.43	0.67^*^	11.87	0.43	11.98	0.41
Illinois Agility Test (s)	15.34	0.36	15.08	0.36^*^	15.83	0.94	15.62	0.73
Depth vertical jump (cm)	44.8	9.5	48.8	11.1^*^	40.62	4.6	40.55	7.1
MVC (Nm)	304.97	65.1	327.99	69.7^*^	282.10	41.81	287.13	47.0
